# DHODH regulates trophoblast fusion via IFITM-reduced plasma membrane fluidity: Implications for hypertensive disorders of pregnancy

**DOI:** 10.1016/j.isci.2026.116163

**Published:** 2026-06-08

**Authors:** Kanoko Yoshida, Kazuya Kusama, Junya Kojima, Yu Kawaguchi, Kaito Suzuki, Tomoka Shimooki, Atsuya Tsuru, Mikihiro Yoshie, Masanori Ono, Hirotaka Nishi, Kiyoko Kato, Kazuhiro Tamura

**Affiliations:** 1 Laboratory of Endocrine Pharmacology, Tokyo University of Pharmacy and Life Sciences, Tokyo 192-0392, Japan; 2 Department of Obstetrics and Gynecology, Tokyo Medical University, Tokyo 160-8402, Japan; 3 Department of Obstetrics and Gynecology, Graduate School of Medical Sciences, Kyushu University, Fukuoka 812-8582, Japan

**Keywords:** Reproductive medicine, Molecular biology, Cell biology

## Abstract

Hypertensive disorders of pregnancy (HDPs) affect 5%–10% of pregnant women and, in the absence of established therapies, remain a leading cause of maternal and perinatal mortality. HDPs have been associated with disruption of cytotrophoblast fusion into syncytiotrophoblasts, a process essential for placental development. Here, we identified altered expression of mitochondrial dihydroorotate dehydrogenase (DHODH) and interferon-induced transmembrane proteins (IFITMs), using HDP placentas, and investigated their functional roles in trophoblast fusion and membrane dynamics. In trophoblast cells, the inhibition of DHODH led to upregulated expression of IFITM1–3 through transcription factor IRF1 and suppression of syncytialization. IFITMs also increased the proportion of saturated fatty acids, thereby decreasing plasma membrane fluidity. Furthermore, IFITM2 increased the soluble fms-like tyrosine kinase-1/placental growth factor (sFlt1/PlGF) ratio, a key biomarker of HDP severity. These results suggest that DHODH deficiency activates IRF1-mediated IFITM2 expression, leading to impaired trophoblast fusion via biophysical remodeling of the membrane and contributing to HDP pathogenesis.

## Highlights

• DHODH inhibition induces IRF1-dependent IFITM1–3 expression
• IFITMs suppress trophoblast syncytialization and membrane fluidity
• IFITMs increase saturated lipids and reduce plasma membrane dynamics
• IFITM2 elevates sFlt1/PlGF ratio linked to HDP severity

## Graphical abstract

**Figure:**
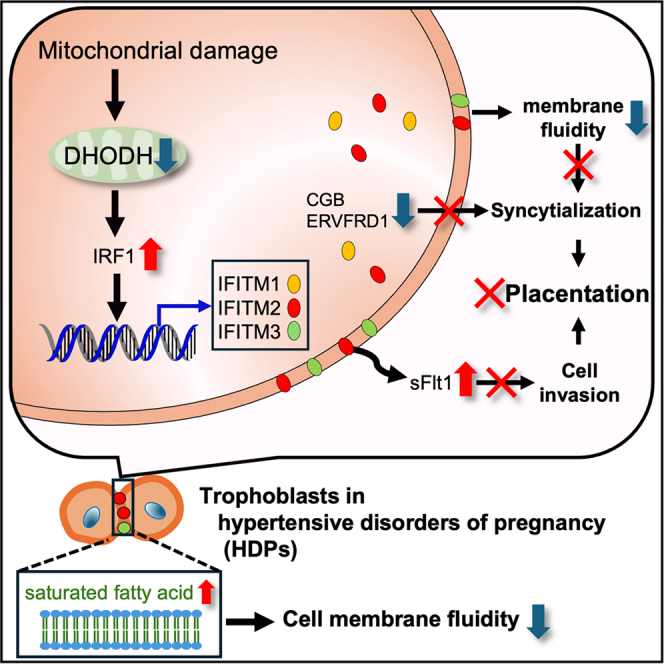

## Introduction

Hypertensive disorders of pregnancy (HDPs) are clinically defined as new-onset hypertension, with proteinuria occurring after 20 weeks of gestation. Although the prevalence of these conditions varies with ethnicity, age, and healthcare access, HDPs affect approximately 5%–10% of pregnancies and are classified into four categories: chronic hypertension, gestational hypertension, preeclampsia, and preeclampsia superimposed on chronic hypertension.^1^^,^^2^^,^^3^ According to a WHO review, hypertensive disorders are responsible for approximately 16% of maternal deaths in high-income countries.^4^ Moreover, HDPs are recognized as a major risk factor for future cardiovascular diseases, highlighting the urgent need for effective therapies and early diagnostic methods.^5^ However, current treatments remain limited to symptomatic management with agents such as labetalol, nifedipine, methyldopa, and hydralazine. Although HDPs are caused primarily by defective placental development due to trophoblast dysfunction,^6^ no definitive therapy has been established to date, as the underlying pathophysiological mechanisms remain poorly understood.^7^^,^^8^

The placenta is a transient organ that plays a critical role in maintaining pregnancy and supporting fetal development. It is primarily composed of trophoblast cells, which differentiate into mononuclear villous cytotrophoblasts (CTBs), multinucleated syncytiotrophoblasts (STs), and extravillous trophoblasts (EVTs).^9^ Specifically, CTBs undergo cell-cell fusion, a process known as syncytialization, to form the multinucleated ST layer. The ST layer facilitates the exchange of oxygen, nutrients, and waste products between the mother and fetus and produces human chorionic gonadotropin (hCG), which is essential for the maintenance of pregnancy. In addition, the ST layer serves as an immunological barrier, protecting the fetus from maternal infections such as cytomegalovirus and Zika virus, as well as from exposure to harmful substances, including various drugs.^10^^,^^11^ EVTs, on the other hand, invade the maternal endometrium and remodel the uterine spiral arteries to increase blood flow to the placenta. This arterial remodeling is promoted by angiogenic factors such as placental growth factor (PlGF) and inhibited by soluble fms-like tyrosine kinase-1 (sFlt1). In placentas from patients with HDPs, circulating levels of sFlt1 are elevated, and the sFlt1/PlGF ratio serves as a marker of HDP severity. These changes are thought to result from impaired trophoblast fusion and invasion.^12^

In cultured human primary trophoblasts and the human trophoblast-derived choriocarcinoma cell line BeWo, elevated intracellular cyclic AMP (cAMP) levels induce the production of hCG and progesterone. This increase also upregulates the expression of the fusogenic proteins syncytin-1 (ERVW1) and syncytin-2 (ERVFRD1), which are encoded by endogenous retroviruses (ERVs), through the action of the transcription factor glial cells missing 1 (GCM1), ultimately promoting trophoblast cell fusion or syncytialization.

Increasing evidence suggests that pregnancy complications may involve elevated oxidative stress and reduced antioxidant capacity, both of which contribute to the inhibition of trophoblast syncytialization. In addition, placentas from patients with HDPs exhibit mitochondrial dysfunction, including reduced mitochondrial DNA content and diminished complex IV activity.^13^^,^^14^ Mitochondria generate adenosine triphosphate (ATP) via oxidative phosphorylation, but they can also contribute to cellular dysfunction through aberrant calcium handling and the overproduction of reactive oxygen species (ROS) during energy metabolism. Mitochondrial dysfunction, particularly ROS accumulation, is thus a major driver of oxidative stress. We previously demonstrated that mitochondrial dysfunction is exacerbated during syncytialization, resulting in increased ROS levels and oxidative stress, which, in turn, suppresses the syncytialization process. Furthermore, antioxidant treatment was found to increase the expression of mitochondrial functional markers, promote mitophagy, increase the mitochondrial membrane potential, and reduce the levels of ROS and oxidative stress markers during syncytialization.^15^ These findings suggest that mitochondrial dysfunction may lead to decreased intracellular cAMP levels via impaired ATP production, potentially disrupting syncytialization.^15^ However, the mechanistic link between mitochondrial dysfunction and HDPs remains poorly understood.

Dihydroorotate dehydrogenase (DHODH), an oxidoreductase localized to the mitochondrial inner membrane, plays a central role in *de novo* pyrimidine biosynthesis. In addition to its metabolic functions, DHODH has been implicated in the suppression of mitochondrial lipid peroxidation and in protection against ferroptosis. Notably, DHODH inhibition has been shown to induce interferon (IFN)-stimulated gene expression, independent of the canonical IFN signaling pathways.

In the present study, we observed that DHODH expression is altered in placentas from HDP patients. However, the physiological and pathological roles of DHODH in placental function remain largely unknown. We, therefore, hypothesized that mitochondrial DHODH contributes to trophoblast syncytialization. To test this hypothesis, we conducted RNA sequencing (RNA-seq) analysis of the placental tissues from patients with HDPs to identify mitochondria-related factors that may be involved in HDP pathogenesis.

## Results

### Increased IFITM and decreased DHODH expression characterize the placenta in the context of HDPs

**Figure 1:**
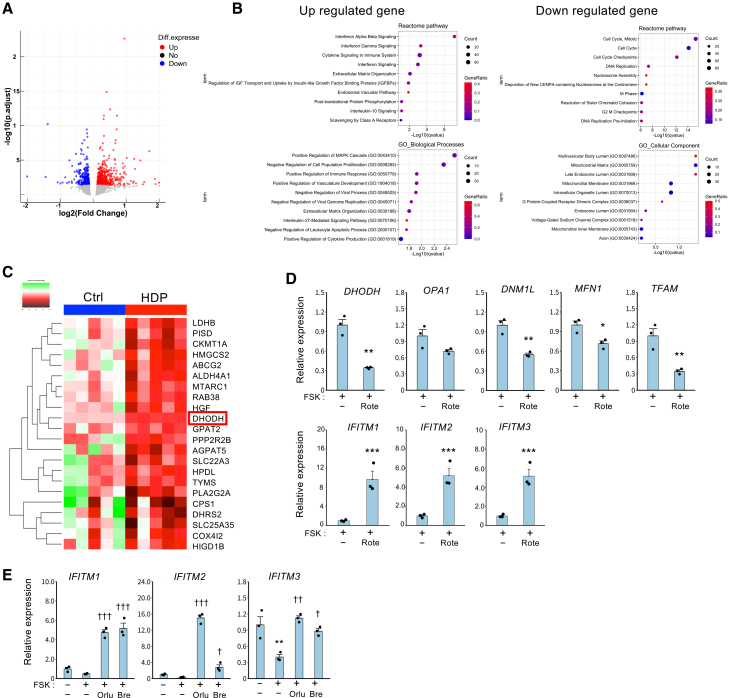
Increased IFITM and decreased DHODH expression characterize the placenta in the context of HDPs (A) Volcano plot of transcriptomic changes identified by RNA-seq. Transcripts highlighted in red or blue were significantly altered (*q* value < 0.05). (B) Differentially expressed genes were classified by functional enrichment analysis using the Reactome pathway database and Gene Ontology (GO) biological processes or cellular components. (C) Heatmap of mitochondria-related genes downregulated in the placenta in the context of HDPs. Genes with higher expression are shown in green, and those with lower expression are shown in red. Ctrl: premature delivery, *n* = 5; HDP, *n* = 5. (D) Expression of DHODH, OPA1, DNM1L, MFN1, TFAM, and IFITM1-3 in trophoblast BeWo cells treated with forskolin (FSK, 2.5 μM) and rotenone (Rote, 50 nM) for 48 h. GAPDH was used as a reference gene. The data are presented as the mean ± SEM from three independent experiments. ∗*p* < 0.05, ∗∗*p* < 0.01 vs. FSK alone (Tukey’s test). (E) Expression of IFITMs in BeWo cells treated with FSK (2.5 μM), orludodstat (Orlu, 1 nM), or brequinar (Bre, 25 nM) for 48 h. GAPDH was used as a reference gene. The data are presented as the mean ± SEM from three independent experiments. ∗∗*p* < 0.01 vs. Ctrl; †*p* < 0.05, ††*p* < 0.01, †††*p* < 0.001 vs. FSK alone (Tukey’s test).

To identify genes associated with HDPs, we performed RNA-seq analysis of placental tissues collected at 22–28 weeks of gestation from HDP patients and premature delivery. A total of 549 downregulated genes and 859 upregulated genes were identified in placentas from HDP patients compared to those from premature delivery (Figure 1A). Pathway enrichment analysis of the differentially expressed genes using the Reactome pathway database and Gene Ontology (GO) database revealed significant activation of IFN-related signaling and the immune response among the upregulated genes. In contrast, mitochondria-related terms were enriched among the downregulated genes in placentas from HDP patients (Figure 1B). We focused on interferon-induced transmembrane proteins (IFITMs), which are included in IFN-related signaling pathways (Figure S1). IFITMs are a family of transmembrane proteins induced by IFNs. In humans, IFITM genes are expressed in various tissues, and in particular, IFITM1, IFITM2, and IFITM3 have been implicated in embryonic development, cell adhesion, tumorigenesis, signal transduction, and antiviral responses.^16^^,^^17^^,^^18^^,^^19^ IFITMs have been reported to block the formation of fusion pores following endosome hemifusion, either by directly altering the physical properties of the plasma membrane or by modifying its lipid composition.^16^^,^^20^ This effect is thought to be associated with IFITM-induced reductions in host cell membrane fluidity.^21^ Although IFITMs have been shown to suppress syncytialization and induce placental abnormalities in mouse models, the underlying mechanisms remain poorly understood.^22^^,^^23^^,^^24^ Because DHODH inhibition has been reported to induce IFN-stimulated gene expression independent of canonical IFN signaling pathways,^25^ DHODH was of particular interest among the downregulated genes identified in placentas from HDP patients (Figure 1C). Treatment with rotenone (Rote), a mitochondrial complex I inhibitor, led to a reduction in the expression of several mitochondrial markers, including *DHODH*; *OPA1*, a GTPase that is localized in the mitochondrial inner membrane; *DNM1L,* a mitochondrial fission marker; *MFN1*, which maintains mitochondrial morphology; *TFAM*, a mitochondrial DNA replication and repair factor; and IFITM1-3 (Figure 1D). To evaluate the effect of DHODH inhibition on *IFITM* expression, BeWo cells were treated with the DHODH inhibitors orludodstat (Orlu) and brequinar (Bre). Both the Orlu and Bre treatments increased the expressions of *IFITM1*, *IFITM2*, and *IFITM3* (Figure 1E). Consistently, DHODH inhibition by Orlu and Bre also increased the expression of IFITM1, IFITM2, and IFITM3 in trophoblast stem cells (Figure S2).

### Inhibition of DHODH increases IFITM expression and suppresses syncytialization

**Figure 2:**
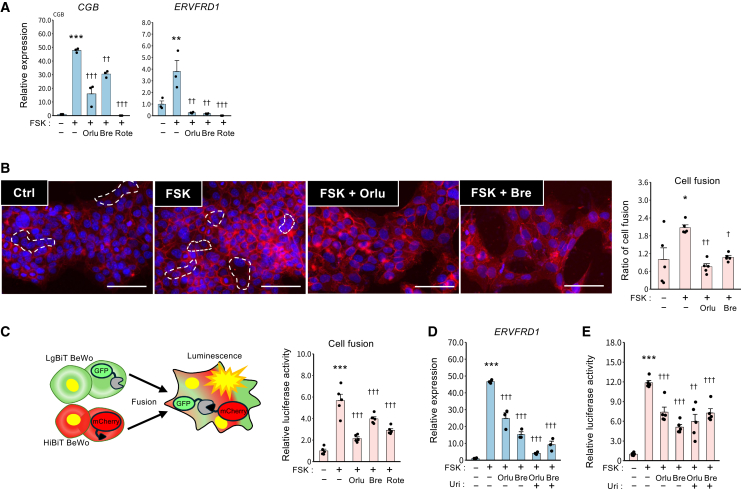
Inhibition of DHODH suppresses syncytialization (A–C) BeWo cells were treated with FSK (2.5 μM), Orlu (1 nM), Bre (25 nM), or Rote (50 nM) for 48 h. (A) Expression of the syncytialization markers CGB and ERVFRD1 was assessed; GAPDH was used as the reference gene. The data are presented as the mean ± SEM from three independent experiments. ∗∗*p* < 0.01, ∗∗∗*p* < 0.001 vs. Ctrl; ††*p* < 0.01, †††*p* < 0.001 vs. FSK alone (Tukey’s test). (B) The cells were immunostained with anti-E-cadherin (green, cell membrane) and DAPI (blue, nuclei) to visualize syncytialization. A representative image from five independent experiments is shown; syncytialized cells are indicated by dashed outlines (left). Scale bars, 100 μm. The number of syncytialized cells was quantified in five randomly selected fields per experiment (right). The data are presented as ratios to Ctrl and are expressed as the mean ± SEM. ∗*p* < 0.05 vs. Ctrl; †*p* < 0.05, ††*p* < 0.01 vs. FSK alone (Tukey’s test). (C) Luciferase activity was measured to evaluate cell fusion. Data from five independent experiments are shown as the mean ± SEM. ∗∗∗*p* < 0.001 vs. Ctrl; †††*p* < 0.001 vs. FSK alone (Tukey’s test). (D and E) BeWo cells were treated with FSK (2.5 μM), Orlu (1 nM), Bre (25 nM), or uridine (Uri, 100 μM) for 48 h. (D) The expression of ERVFRD1 was assessed by qPCR. GAPDH was used as the reference gene. The data are shown as the mean ± SEM from three independent experiments. ∗∗∗*p* < 0.001 vs. Ctrl; †††*p* < 0.001 vs. FSK alone (Tukey’s test). (E) Luciferase activity was measured to assess cell fusion. The data are presented as the mean ± SEM from three independent experiments. ∗∗∗*p* < 0.001 vs. Ctrl; ††*p* < 0.01, †††*p* < 0.001 vs. FSK alone (Tukey’s test).

We next investigated whether DHODH plays a role in regulating *IFITM* expression and trophoblast syncytialization. Orlu, Bre, and Rote treatments also decreased the expression of syncytialization markers such as CGB and ERVFRD1 (Figure 2A). Morphological analysis further revealed that both inhibitors significantly reduced the number of syncytialized cells (Figure 2B). In a complementary fusion assay, BeWo cells expressing GFP-LgBiT and mCherry-HiBiT were co-cultured and treated with Orlu, Bre, or Rote. Forskolin (FSK)-induced syncytialization was significantly suppressed by all three treatments (Figure 2C). To determine whether the observed suppression of syncytialization induced by DHODH inhibition was due to impaired pyrimidine synthesis, we supplemented the cultures with uridine, the end product of this pathway. However, uridine supplementation did not affect *ERVFRD1* expression (Figure 2D), nor did it restore cell fusion in GFP-LgBiT- or mCherry-HiBiT-expressing BeWo cells (Figure 2E). These results indicate that pharmacological inhibition of DHODH with Orlu, Bre, or Rote suppressed syncytialization independent of the *de novo* pyrimidine synthesis pathway.

### NR2F1 and ZFN554 regulate DHODH expression

**Figure 3:**
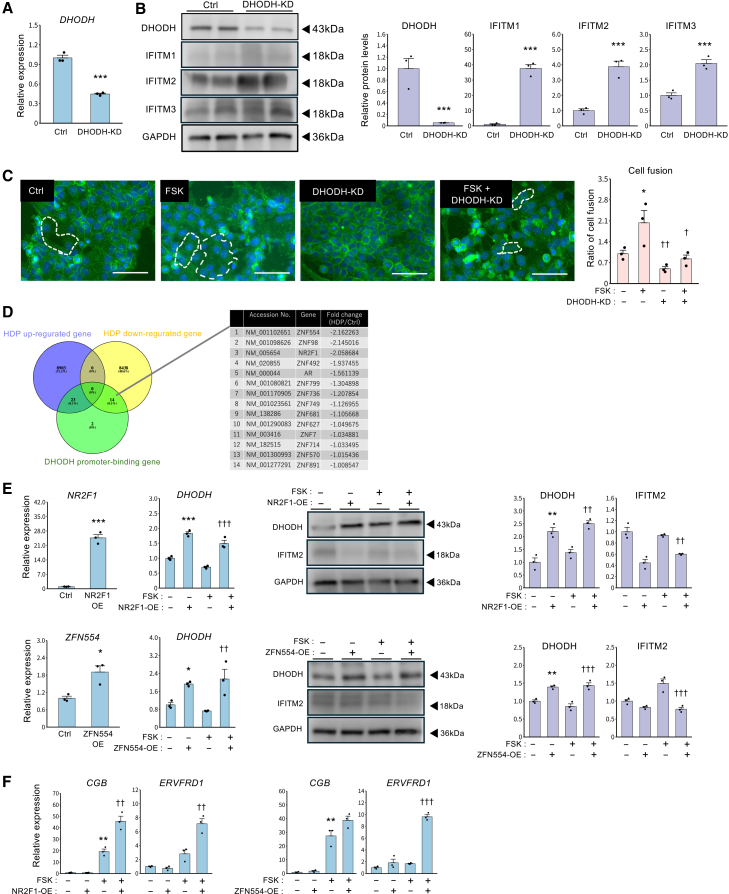
NR2F1 and ZFN554 regulate DHODH expression (A) Expression of DHODH in DHODH knockdown (DHODH-KD) BeWo cells. The data are presented as the mean ± SEM from three independent experiments. ∗∗∗*p* < 0.001 vs. Ctrl (Student’s *t* test). (B) Immunoblotting for DHODH, IFITM1, IFITM2, and IFITM3. GAPDH was used as a loading control. Representative data from three independent experiments are shown. The graph shows DHODH, IFITM1, IFITM2, and IFITM3 levels normalized to GAPDH levels from three independent experiments. ∗∗∗*p* < 0.001 vs. Ctrl (Student’s *t* test). Values represent the mean ± SEM. (C) BeWo and DHODH-KD BeWo cells were treated with FSK (2.5 μM) for 48 h. Cells were immunostained with anti-E-cadherin (green, cell membrane) and DAPI (blue, nuclei) to visualize syncytialization. Representative images from three independent experiments are shown; syncytialized cells are outlined with dashed lines (left). Scale bars, 100 μm. The number of syncytialized cells was quantified in three randomly selected fields per experiment (right). The data are shown as ratios relative to the control and are presented as the mean ± SEM. ∗*p* < 0.05 vs. Ctrl; †*p* < 0.05, ††*p* < 0.01, vs. FSK alone (Tukey’s test). (D) Venn diagram analysis of transcriptional regulators. Blue: genes upregulated in placental tissue in the context of HDPs; yellow: genes downregulated in placental tissue in the context of HDPs; green: genes predicted to encode proteins with potential binding sites in the DHODH promoter region. (E) Expression of NR2F1, DHODH, and ZFN554 in BeWo cells overexpressing NR2F1 or ZFN554 (NR2F1-OE or ZFN554-OE), as determined by qPCR (left) and immunoblotting (right). The data are shown as the mean ± SEM from three independent experiments. ∗*p* < 0.05, ∗∗∗*p* < 0.001 vs. Ctrl; ††*p* < 0.01, †††*p* < 0.001 vs. FSK (Student’s *t* test or Tukey’s test). GAPDH was used as a loading control. Representative data from three independent experiments are shown. The graph shows DHODH and IFITM2 levels normalized to GAPDH levels from three independent experiments. ∗*p* < 0.05, ∗∗*p* < 0.01 vs. Ctrl (Tukey’s test). Values represent the mean ± SEM. (F) Expression of syncytialization markers CGB and ERVFRD1 in NR2F1-OE or ZFN554-OE BeWo cells treated with FSK (2.5 μM), as measured by qPCR. The data are presented as the mean ± SEM from three independent experiments. ∗∗*p* < 0.01 vs. Ctrl; ††*p* < 0.01, †††*p* < 0.001 vs. FSK alone (Tukey’s test).

Because pharmacological inhibition of DHODH increased *IFITM* expression and suppressed syncytialization, we next examined whether DHODH knockdown (KD) (Figure 3A) would produce similar effects, and we found that DHODH-KD resulted in elevated *IFITM* expression (Figure 3B). Furthermore, morphological analysis revealed a significant reduction in the number of syncytialized cells upon DHODH-KD (Figure 3C). To investigate the upstream regulatory mechanisms controlling DHODH expression in trophoblasts, we analyzed the transcription factors that may be involved in DHODH regulation. We compared 39 transcription factor genes predicted to bind within 3 kb of the DHODH promoter with genes that were differentially expressed in placental tissues from HDP patients. This analysis identified 14 genes that were predicted both to bind to the DHODH promoter and to be downregulated in HDPs. Among these genes, *NR2F1* (nuclear receptor subfamily 2 group f member 1) and *AR* (androgen receptor) have previously been reported to bind to the DHODH promoter, while the remaining 12 were predicted on the basis of sequence motifs. *NR2F1* and *ZNF554* (zinc-finger protein 554) presented the most pronounced fold changes in expression (Figure 3D). Consequently, NR2F1 (a nuclear receptor-type TF) and ZNF554 (a zinc finger-type TF) presented the most pronounced fold changes in expression among their respective groups (Figure 3D). To evaluate the effects of *NR2F1* and *ZFN554* overexpression (OE) on *DHODH* and *IFITM* expressions, as well as syncytialization, we overexpressed these factors in BeWo cells (Figures 3E and 3F). NR2F1-OE and ZFN554-OE increased *DHODH* expression, decreased *IFITM2* expression (Figure 3E), and increased the expression of syncytialization markers (Figure 3F). These results indicate that DHODH expression is regulated by NR2F1 and ZNF554 and that its inhibition in turn impaired trophoblast syncytialization.

### DHODH regulates IFITM expression via IRF1

**Figure 4:**
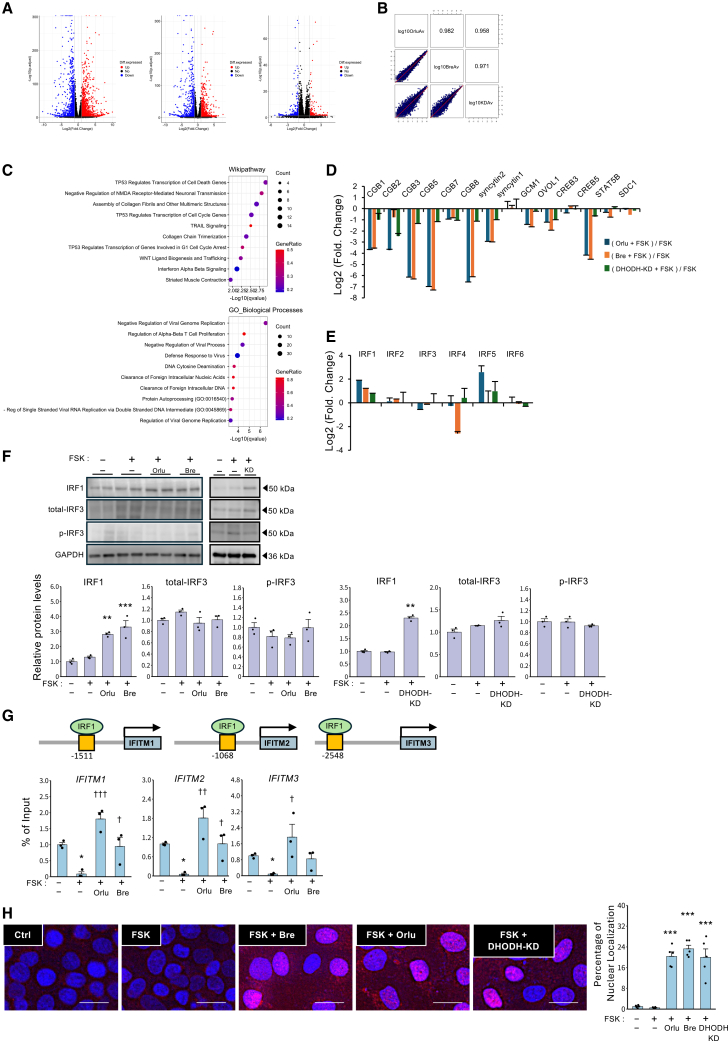
DHODH regulates IFITM expression via IRF1 (A–H) BeWo cells or DHODH-KD BeWo cells were treated with FSK (2.5 μM), Orlu (1 nM), or Bre (25 nM) for 48 h. (A) Volcano plot showing transcriptomic changes identified by RNA-seq. Transcripts highlighted in red or blue were considered differentially expressed, as indicated by an expression change ≥2-fold (*p* < 0.05). (B) Correlation analysis of RNA-seq data from Orlu-, Bre-treated, and DHODH-KD cells. (C) Differentially expressed genes were classified by functional enrichment analysis using the Wiki pathway database and GO biological processes or cellular components. (D) RNA-seq was used to evaluate the expression levels of genes associated with syncytialization. (E) RNA-seq was used to evaluate the expression levels of IRF family genes. (F) Immunoblotting for IRF1, total IRF3, and *p*-IRF3. GAPDH was used as a loading control. Representative data from three independent experiments are shown. The graph shows the total IRF3 and *p*-IRF3 levels normalized to GAPDH levels from three independent experiments. ∗∗*p* < 0.01, ∗∗∗*p* < 0.001 vs. Ctrl (Tukey’s test). Values represent the mean ± SEM. (G) ChIP assay showing IRF1 binding to upstream regulatory regions (up to 3 kbp) of the IFITM1, IFITM2, and IFITM3 loci in BeWo cells treated with FSK alone (2.5 μM) for 48 h. ∗*p* < 0.05 vs. Ctrl; †*p* < 0.05, ††*p* < 0.01, †††*p* < 0.001 vs. FSK alone (Tukey’s test). (H) Immunofluorescence staining of IRF1 (red). Nuclei were counterstained with DAPI (blue). Scale bars, 5 μm. The graph shows the number of staining cells from three independent experiments. Values represent the mean ± SEM. ∗∗∗*p* < 0.001 vs. FSK.

To investigate the effects of Orlu, Bre, and DHODH-KD on gene expression in BeWo cells, we performed RNA-seq analyses. Pairwise comparisons among the Orlu, Bre, and DHODH-KD treatments revealed strong correlations, with correlation coefficients of approximately 0.9 (Figures 4A and 4B). These results indicate that the differentially expressed genes induced by Orlu closely resemble those induced by Bre treatment or DHODH-KD. Consequently, we focused on the Orlu-treated group for the signaling pathway and GO enrichment analyses of the upregulated genes. These analyses revealed significant alterations in IFN-related signaling and the genes associated with viral infection (Figure 4C). Notably, several syncytialization markers were downregulated in the RNA-seq dataset (Figure 4D). On the basis of previous studies indicating that members of the interferon regulatory factor (IRF) family regulate *IFITM* expression, we examined the expression of IRF family transcription factors in our RNA-seq data.^26^ Among these genes, IRF1 expression was upregulated in response to Orlu, Bre, and DHODH-KD (Figure 4E). Previous studies have shown that mitochondrial damage leads to the release of mitochondrial DNA, which can bind to ZBP1 and increase IRF1 transcription in chondrocytes.^27^ In our study, the levels of IRF1 were elevated after Orlu, Bre, and DHODH-KD treatments, while the levels of phosphorylated IRF3 (*p*-IRF3) remained unchanged (Figure 4F). To determine whether IRF1 directly regulates *IFITM* expression by binding upstream of transcription start sites, we performed chromatin immunoprecipitation (ChIP) assays. These assays revealed that IRF1 binds to the promoter regions of *IFITM1*, *IFITM2*, and *IFITM3* in BeWo cells treated with Orlu or Bre (Figure 4G). Furthermore, Orlu, Bre, and DHODH-KD treatments promoted the nuclear translocation of IRF1 (Figure 4H). Collectively, these findings demonstrate that DHODH inhibition selectively activates IRF1, which directly drives IFITM transcription independently of IRF3.

### IFITM regulates trophoblast syncytialization

**Figure 5:**
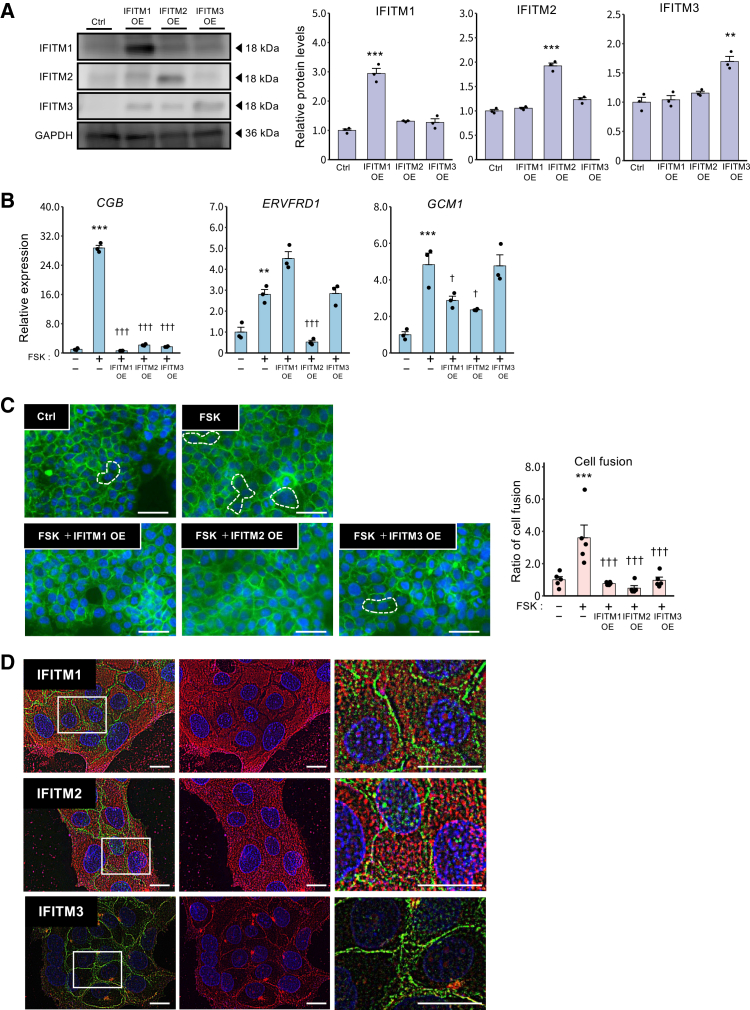
IFITM regulates trophoblast syncytialization (A) Immunoblot analysis of lysates from IFITM1OE-, IFITM2-OE, or IFITM3-OE BeWo cells; GAPDH was used as a loading control. Representative data from three independent experiments are shown. The graph shows IFITM1, IFITM2, and IFITM3 levels normalized to GAPDH levels from three independent experiments. ∗∗*p* < 0.01, ∗∗∗*p* < 0.001 vs. Ctrl (Tukey’s test). Values represent the mean ± SEM. (B) Expression of syncytialization markers CGB, ERVFRD1, and GCM1 in IFITM-OE BeWo cells treated with FSK (2.5 μM) for 48 h. GAPDH was used as the reference gene. The data are presented as the mean ± SEM from three independent experiments. ∗∗*p* < 0.01, ∗∗∗*p* < 0.001 vs. Ctrl; †*p* < 0.05, †††*p* < 0.001 vs. FSK (Tukey’s test). (C) Cells were immunostained with anti-E-cadherin (green, cell membrane) and DAPI (blue, nuclei) to visualize syncytialized cells. A representative image from five independent experiments is shown; fused cells are indicated by dashed outlines (top). Scale bars, 100 μm. The number of syncytialized cells was quantified in five randomly selected fields per experiment (bottom). The data are presented as ratios relative to the control and are expressed as the mean ± SEM. ∗∗∗*p* < 0.001 vs. Ctrl; †††*p* < 0.001 vs. FSK (Tukey’s test). (D) Immunofluorescence-based localization of IFITM1, IFITM2, and IFITM3 (red). Nuclei were counterstained with DAPI (blue), and cell membranes were visualized using E-cadherin (green). Scale bars, 20 μm.

We next investigated the role of IFITMs in the syncytialization of BeWo cells overexpressing each IFITM subtype (Figure 5A). The overexpression of any IFITM subtype reduced CGB expression and cell fusion, whereas the expression of ERVFRD1 and GCM1 was specifically decreased in IFITM2-OE cells (Figures 5B and 5C). To elucidate the mechanism by which IFITMs inhibit syncytialization, we examined their subcellular localization in BeWo cells. Immunofluorescence analysis demonstrated that IFITM2 and IFITM3 were predominantly localized to the plasma membrane, in contrast to IFITM1 (Figure 5D). These results indicated that IFITM2 and IFITM3, primarily localized at the plasma membrane, may inhibit functional and morphological syncytialization.

### Reduced plasma membrane fluidity in IFITM2- or IFITM3-OE BeWo cells

**Figure 6:**
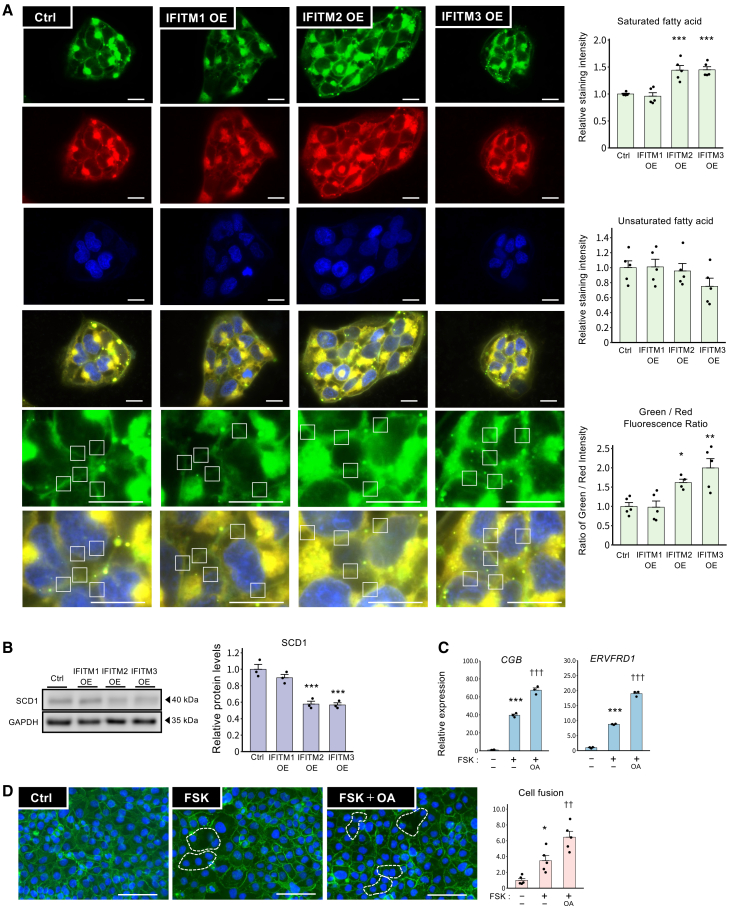
Reduced plasma membrane fluidity in IFITM2-OE and IFITM3-OE BeWo cells (A) Fluorescence micrographs showing the localization of saturated fatty acids in IFITM1-OE, IFITM2-OE, and IFITM3-OE BeWo cells. The cells were stained with a solvatochromic fluorescent dye (green, saturated fatty acids; red, unsaturated fatty acids) and DAPI (blue, nuclei). A representative image from three independent experiments is shown (left). Scale bars, 20 μm. The graph shows the fluorescence intensity of saturated fatty acids measured within the white boxed areas (right). Values represent the mean ± SEM. ∗∗∗*p* < 0.001 vs. Ctrl (Tukey’s test). (B) Immunoblotting analysis of SCD1 protein levels in IFITM1-OE, IFITM2-OE, and IFITM3-OE BeWo cells. GAPDH was used as a loading control. Representative data from three independent experiments are shown. The graph shows IFITM1, IFITM2, and IFITM3 levels normalized to GAPDH levels from three independent experiments. ∗∗*p* < 0.01, ∗∗∗*p* < 0.001 vs. Ctrl (Tukey’s test). Values represent the mean ± SEM. (C and D) BeWo cells were treated with FSK (2.5 μM) and oleic acid (OA, 5 μM) for 48 h. (C) Expression levels of syncytialization markers CGB and ERVFRD1 were assessed by qPCR. GAPDH was used as the reference gene. The data are shown as the mean ± SEMs from three independent experiments. ∗∗∗*p* < 0.001 vs. Ctrl; †††*p* < 0.001 vs. FSK (Tukey’s test). (D) Cells were immunostained with E-cadherin (green, cell membrane) and DAPI (blue, nuclei) to visualize syncytialized cells. A representative image from five independent experiments is shown; the fused cells are outlined with dashed lines (left). Scale bars, 100 μm. The number of syncytialized cells was quantified in five randomly selected fields per experiment (right). The data are expressed as ratios relative to Ctrl and are presented as the mean ± SEM. ∗*p* < 0.05 vs. Ctrl; ††*p* < 0.01 vs. FSK (Tukey’s test).

IFITM2 is localized to the plasma membrane in BeWo cells and inhibits syncytialization. However, the detailed mechanisms by which IFITM2 suppresses syncytialization remain unknown. Lipid composition influences the biophysical properties of cell membranes; unsaturated fatty acids, in particular, increase membrane fluidity. Membrane fluidity plays a crucial role in various membrane-associated cellular processes, such as cell fusion, including the function of membrane proteins.^28^ We assessed cell membrane fluidity in IFITM-OE BeWo cells by quantifying the levels of saturated fatty acids within the membrane. IFITM2-OE or IFITM3-OE significantly increased the levels of saturated fatty acids on the plasma membrane, whereas unsaturated fatty acids showed no significant change (Figure 6A). In addition, stearoyl-CoA desaturase 1 (SCD1) increases plasma membrane fluidity by promoting the synthesis of unsaturated fatty acids.^29^ IFITM2-OE and IFITM3-OE reduced the expression of SCD1 (Figure 6B). To further determine whether cell membrane fluidity influences syncytialization, we treated BeWo cells with oleic acid (OA), an unsaturated fatty acid, and evaluated functional and morphological syncytialization. OA treatment enhanced the expression of *CGB* and *ERVFRD1* and inhibited cell fusion (Figures 6C and 6D). In trophoblasts, IFITM2/3-OE decreased SCD1 expression and increased saturated fatty acids in the plasma membrane, thereby reducing membrane fluidity and inhibiting syncytialization.

### IFITM alters the expression of angiogenic markers and inhibits the invasion of EVTs

**Figure 7:**
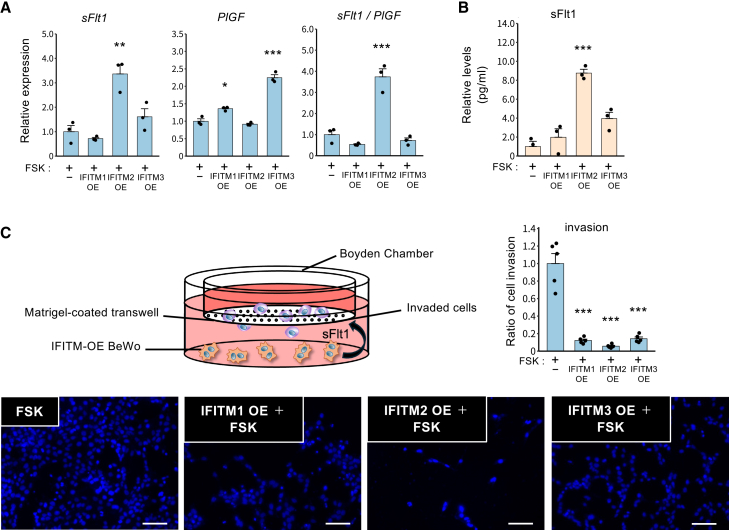
IFITM alters angiogenic markers and inhibits EVT invasion (A) Expression of HDP-related markers sFlt1 and PlGF in IFITM1-OE, IFITM2-OE, and IFITM3-OE BeWo cells treated with FSK (2.5 μM), as measured by qPCR. GAPDH was used as the reference gene. The corresponding sFlt1/PlGF ratio is shown on the right. The data are presented as the mean ± SEM from three independent experiments. ∗*p* < 0.05, ∗∗*p* < 0.01, ∗∗∗*p* < 0.001 vs. FSK (Tukey’s test). (B) Culture media were analyzed by ELISA to measure secreted sFlt1 levels. The data are shown as the mean ± SEM from three independent experiments. ∗∗∗*p* < 0.001 vs. FSK (Tukey’s test). (C) EVT HTR8/SVneo cell lines (HTR8) were seeded in the upper chambers of Matrigel-coated transwells, and IFITM-OE BeWo cells were pre-seeded in the lower chambers prior to the invasion assay. After 48 h of co-culture, the effect of IFITM-OE BeWo cell-derived sFlt1 on HTR8 invasion was evaluated (top left). Nuclei were stained with DAPI (blue). Representative images are shown (bottom). The numbers of cells that passed through the transmembrane were counted and are presented as the mean ± SEM of five independent experiments (top right). ∗∗∗*p* < 0.001 vs. FSK (Tukey’s test).

We next examined the impact of IFITM-OE on the expression levels of sFlt1 and PlGF, which are implicated in severe HDPs. Accordingly, we measured the expression of HDP-associated markers and calculated the sFlt1/PlGF ratio. IFITM2-OE increased sFlt1 levels and the sFlt1/PlGF ratio (Figure 7A). Secretion of the sFlt1 protein was also increased in IFITM2-OE BeWo cells (Figure 7B). Furthermore, we investigated whether sFlt1 secreted from IFITM-OE BeWo cells affects the invasion capacity of EVTs, using a co-culture system. Co-culture with IFITM-OE BeWo cells significantly suppressed the invasion of EVT HTR8/SVneo cell (HTR8) (Figure 7C). In syncytializing trophoblasts, IFITM2-OE increased sFlt1 expression and secretion and consequently suppressed EVT invasion in a co-culture system.

### Differential expression of IFITM and DHODH in placental tissues from HDP patients

**Figure 8:**
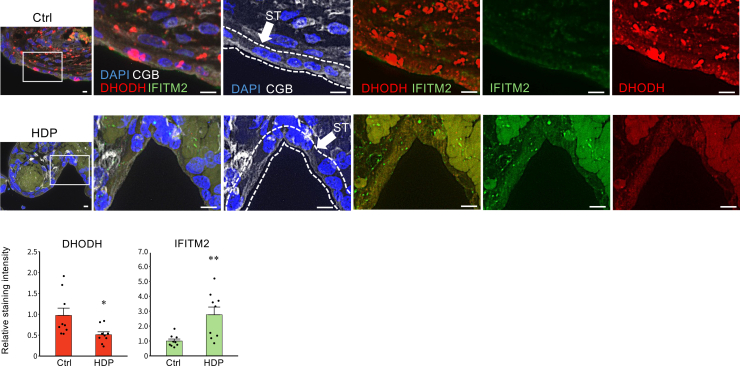
Differential expression of IFITM2 and DHODH in placental tissues from HDP patients Placental sections from the premature delivery (Ctrl, *n* = 3), and HDP (*n* = 3) were immunostained with anti-IFITM2 antibody (green), anti-DHODH antibody (red), anti-hCGB antibody (white, syncytiotrophoblasts [STs]), and DAPI (blue) to label nuclei. For each group, images in the right are higher-magnification views of the ST regions shown on the left. A representative image from three independent experiments is shown. Scale bars, 20 μm. ∗*p* < 0.05 ∗∗*p* < 0.01 vs. Ctrl (Student’s *t* test).

Because IFITM2, a plasma membrane-localized protein, has been shown to reduce membrane fluidity and suppress both *ERVFRD1* expression and cell fusion, we next examined its expression and localization in human placental tissues. To characterize the expression patterns of DHODH and IFITM2, placental tissues from women with HDPs or premature delivery were subjected to immunostaining. The ST layer was identified by positive staining for CGB and the presence of multinucleated cells, while the CGB-negative regions were defined as the CT layer. Compared with placentas from premature delivery, placentas from HDP patients presented reduced DHODH expression and increased IFITM2 expression in the ST layer (Figures 8 and S3).

## Discussion

**Figure 9:**
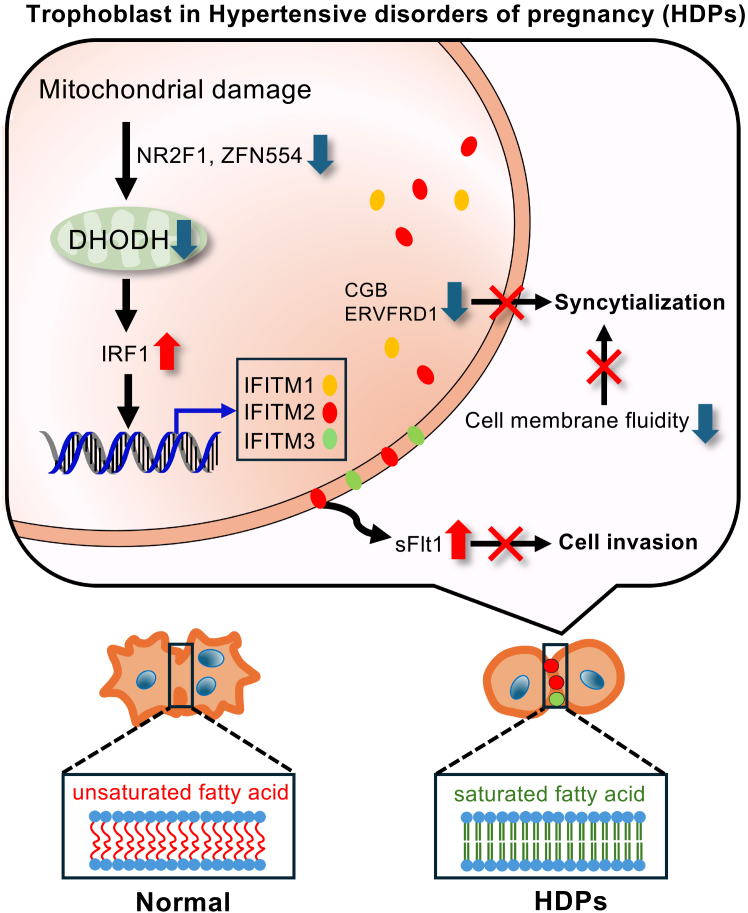
Proposed mechanism of DHODH-IFITM signaling in the placenta in the context of HDPs Schematic of the proposed pathway by which mitochondrial dysfunction alters placental function in HDPs. Mitochondrial damage leads to reduced expression of DHODH, in part, due to downregulation of the transcription factors NR2F1 and ZNF554. Decreased DHODH expression activates IRF1, which upregulates IFITM1, IFITM2, and IFITM3 expressions. Increased levels of IFITM2 and IFITM3 promote the accumulation of saturated fatty acids in the plasma membrane, thereby decreasing membrane fluidity and suppressing trophoblast syncytialization. Furthermore, IFITM2 overexpression enhances the secretion of the antiangiogenic factor sFlt1 and inhibits EVT invasion, contributing to the development of HDPs.

This study demonstrated that mitochondrial dysfunction and DHODH inhibition in trophoblasts lead to increased expression of IFITM via the activation of IRF1. Elevated IFITM levels increase the accumulation of saturated fatty acids in the trophoblast plasma membrane, resulting in reduced membrane fluidity and subsequent inhibition of syncytialization. Furthermore, IFITM2-OE increased the secretion of sFlt1 and modulated the invasion capacity of EVTs. These findings show that reduced mitochondrial DHODH expression in trophoblasts may contribute to the pathogenesis of HDPs (Figure 9).

We observed significant activation of IFN-related signaling pathways; upregulation of ISGs such as IFITM1, IFITM2, and IFITM3; and downregulation of mitochondria-related genes in placental tissues from patients with HDPs. Interestingly, the expression levels of IFNs themselves remained unchanged in placentas from HDP patients. This observation aligns with previous reports indicating that ISG expression can be induced by intracellular stress, such as ER stress and mitochondrial dysfunction, independent of IFN signaling.^30^^,^^31^ Notably, the release of mtDNA has been shown to activate the cGAS-STING-TBK1 pathway, thereby triggering ISG induction in autoimmune disease models.^32^^,^^33^ These findings suggest that similar stress conditions in placentas from HDP patients may drive IFITM expression independently of viral infection. In this study, we demonstrated that treatment with Rote, a mitochondrial complex I inhibitor, led to reduced DHODH expression. Previous studies have shown that DHODH depletion can partially inhibit respiratory chain complex III, reduce the mitochondrial membrane potential, and increase ROS production.^33^ These results suggest a potential feedback loop between mitochondrial dysfunction and DHODH downregulation, which is consistent with prior reports of mitochondrial abnormalities in placentas from HDP patients.^34^ Furthermore, we identified NR2F1 and ZNF554 as candidate transcriptional regulators of DHODH and showed that their overexpression suppresses IFITM2 expression. Notably, ZNF554, a member of the Krüppel-associated box domain zinc-finger protein superfamily, has been reported to be downregulated in placentas from pregnancies complicated by fetal growth restriction. KD of ZNF554 in trophoblast cells was shown to impair the antioxidant capacity and increase ROS production.^35^

We demonstrated that DHODH inhibitors increased IFITM expression and syncytialization via IRF1. Although IRF3 and IRF7 have been extensively studied in the context of antiviral immunity,^36^ the molecular mechanisms governing IRF1 activity remain incompletely understood. Recent studies suggest that IRF1 mediates an antiviral pathway independent of IRF3, IRF5, and IRF7 and contributes to host defense against Dengue virus infection.^37^ Transcriptome analyses have shown that IRF1 regulates the constitutive expression of approximately 300 genes, including antiviral ISGs, and is involved in their epigenetic regulation, indicating the existence of an IFN-independent mechanism of ISG activation.^38^ Previous studies have identified interferon-stimulated response elements (ISREs) in the promoter regions of IFITM genes,^39^ and IRF1 is well known to bind to ISREs and act as a transcriptional activator.^40^ Our RNA-seq analysis revealed that the expression levels of JAK1/2 and STAT1/2 were not significantly altered. Furthermore, classical ISGs such as IRF3, IRF7, IRF9, MX1, and ISG15 also showed no significant changes across Bre- and Orlu-treated or DHODH-KD BeWo cells. These findings suggest that DHODH inhibition regulates IFITM expression through a noncanonical, IRF1-mediated pathway that is independent of the classical JAK-STAT1/2 axis. DHODH plays an essential role in the *de novo* pyrimidine biosynthesis pathway; however, uridine supplementation did not rescue the expression of IFITMs or syncytialization markers, suggesting that DHODH may regulate IFITM expression and trophoblast fusion through a mechanism independent of pyrimidine biosynthesis in trophoblast cells. This DHODH-IRF1-IFITM pathway may be triggered by intracellular stress, particularly mitochondrial dysfunction. In addition to previously reported effects such as increased ROS production and loss of membrane potential,^33^ DHODH impairment has been shown to induce mitochondrial fragmentation,^41^^,^^42^ further reflecting disruption of mitochondrial homeostasis. Excessive ROS production can lead to the release of mtDNA into the cytoplasm, which may subsequently activate IRF1-mediated signaling.^43^

The IFITM family is known to block the early stages of viral replication. Membrane fusion, which plays a critical role in viral entry and infection establishment, is mediated by viral fusion proteins located on the surface of the virion. IFITM1, IFITM2, and IFITM3 inhibit virus-mediated cell-cell fusion, although the degree of inhibition varies depending on the cell type. Furthermore, IFITM1-, IFITM2-, and IFITM3-OE have been shown to alter lipid packing in the plasma membrane, thereby decreasing membrane fluidity and suppressing membrane fusion.^44^ On the basis of this mechanism by which IFITMs suppress host membrane fluidity during viral infection, we hypothesized that they may induce similar changes in trophoblasts to block syncytialization. To test this hypothesis, we analyzed the lipid composition of the plasma membrane. The effect of fatty acids on membrane fluidity is closely related to their molecular shape, which is determined by the degree of saturation in their hydrocarbon chains. Saturated fatty acids make a membrane more rigid, whereas unsaturated fatty acids increase its fluidity.^45^ In our study, IFITM2-OE or IFITM3-OE increased the levels of saturated fatty acids in the plasma membrane, suggesting a reduction in membrane fluidity. The subtype-specific differences in membrane fluidity appear to result from the distinct localization patterns of IFITM1, IFITM2, and IFITM3 in trophoblasts. Compared with IFITM1, IFITM2 was prominently localized at the cell interface. Moreover, IFITM3 expressed at significantly lower levels than the other subtypes in BeWo cells. In addition, the interface localization of IFITMs was not detected in JEG3 cells, a choriocarcinoma cell line that does not undergo syncytialization upon cAMP stimulation. In this study, IFITM2-OE and IFITM3-OE also suppressed SCD1 expression, resulting in increased saturated fatty acid levels and reduced membrane fluidity. While SCD1-mediated lipid desaturation is a well-established mechanism for modulating bilayer fluidity, membrane fluidity is also influenced by the asymmetric distribution of plasma membrane phospholipids. Although the expression levels of flippases and scramblases were not altered in placentas from HDP patients according to our RNA-seq data, further investigation is needed to determine whether phospholipid distribution is affected.^46^^,^^47^^,^^48^^,^^49^ IFITM2-OE has previously been reported to inhibit syncytialization mediated by ERVFRD1, which is consistent with our observation that different IFITM subtypes regulate ERVFRD1 expression in different ways. However, the mechanism underlying the observed reduction in ERVFRD1 expression remains unclear. IFITM2-OE may physically suppress syncytialization by reducing membrane fluidity. Alternatively, altered lipid composition could lead to cleavage of fusion proteins, thereby impairing ERVFRD1 function.^50^ Moreover, reduced cell adhesion via E-cadherin may suppress the transcriptional activation of GCM1, leading to the downregulation of ERVFRD1 expression.^51^

Placental production of sFlt1 has been reported to cause maternal endothelial dysfunction, contributing to HDP symptoms such as hypertension and proteinuria,^52^ as sFlt1 acts as a decoy receptor for VEGF (vascular endothelial growth factor) by reducing the circulating levels of free proangiogenic VEGF. Although placental hypoxia is generally considered a major trigger for increased sFlt1 secretion, the underlying mechanisms remain unclear.^53^ Recent studies have also shown that impaired syncytialization via the PPARγ-GCM1 axis contributes substantially to sFlt1 overproduction.^54^ In this study, IFITM2-OE BeWo cells exhibited increased secretion of sFlt1, and the conditioned media from these cells suppressed the invasion of HTR8/SVneo cells. Because IFITM2-OE significantly inhibited syncytialization, this suppression may have contributed to increased sFlt1 secretion. Although the direct effect of sFlt1 on EVT cells remains to be fully elucidated, the overexpression of human sFlt1 in mice has been reported to impair spiral artery remodeling and reduce trophoblast invasion.^55^ Interestingly, EVT invasion was also inhibited in IFITM1-OE or IFITM3-OE cells, even though these subtypes did not increase sFlt1 secretion. These findings suggest that IFITM-OE may influence EVT behavior through alternative mechanisms. One possibility is that the changes in the membrane lipid composition induced by IFITM-OE alter the secretion of invasion-related proteins, such as matrix metalloproteinases (MMPs) 2 and 9, thereby affecting EVT invasion capacity. Specifically, IFITM-induced alterations in membrane fluidity and lipid packing may impair the exocytosis of MMPs by obstructing the necessary membrane fusion, thereby contributing to the inhibition of EVT invasion.

Collectively, our findings identify the DHODH-IRF1-IFITM axis as a previously unrecognized mechanistic link between mitochondrial dysfunction and placental pathology in HDPs. This study reveals that the axis regulates trophoblast function by altering plasma membrane fluidity, thereby impairing syncytialization and EVT invasion, and provides new mechanistic insight into HDP pathogenesis. In particular, IFITM2-OE in STs may impact not only syncytialization but also EVT invasion, partly through sFlt1 secretion, indicating that IFITMs regulate both key processes of trophoblast function. Nevertheless, future studies using primary trophoblasts and *in vivo* models are essential to validate these findings and to determine whether targeting the DHODH-IRF1-IFITM axis, including IFITMs themselves, could represent a promising therapeutic strategy for the prevention or treatment of HDPs.

### Limitations of the study

This study has several limitations that should be considered when interpreting the findings. BeWo and HTR8/SVneo cells are widely used models of ST differentiation and EVT invasion, respectively, and they may not fully recapitulate the complexity of primary trophoblasts *in vivo*. Validation of the DHODH-IRF1-IFITM axis in primary trophoblasts and animal models is, therefore, important.

## Resource availability

### Lead contact

Requests for further information and resources should be directed to and will be fulfilled by the lead contact, Kazuya Kusama (kusamak@toyaku.ac.jp).

### Materials availability

This study did not generate new unique materials.

### Data and code availability

• Raw RNA-seq data derived from human placental samples and trophoblast cell lines have been deposited at the DNA DataBank of Japan (DDBJ) Sequence Read Archive as DDBJ: DRA021720 and DRA021721 and are publicly available as of the date of publication.
• This study did not generate new code.
• Any additional information required to reanalyze the data reported in this paper is available from the lead contact upon request.

## Acknowledgments

This research was partly supported by 10.13039/501100001691JSPS KAKENHI, Japan (nos. JP 23K18345 and JP24K01913 to K. Kusama and no. JP23KJ1958 to K.Y.).

## Author contributions

K.Y., K. Kusama, J.K., Y.K., K.S., T.S., A.T., M.Y., and M.O. performed the experiments and analyses; K.Y., K. Kusama, and K.T. wrote the main manuscript text; K.Y. and K. Kusama prepared all figures and tables; K.Y., K. Kusama, M.Y., H.N., K. Kato, and K.T. were involved in the planning of the entire experimentation. All authors have reviewed the manuscript.

## Declaration of interests

The authors declare no conflict of interest.

## STAR★Methods

### Key resources table

**Table:** 

REAGENT or RESOURCE	SOURCE	IDENTIFIER
**Antibodies**		

Anti-IFITM1	ProteinTech	Cat# 60074-1-Ig; RRID: AB_2233405
Anti-IFITM2	ProteinTech	Cat# 66137-1-Ig; RRID: AB_2122089
Anti-IFITM3	ProteinTech	Cat# 11714-1-AP; RRID: AB_2295684
Anti-SCD1	ProteinTech	Cat# 28678-1-AP; RRID: AB_ 2923581
Anti-GAPDH	Fujifilm Wako	Cat# 015–25473; RRID: AB_2665526
Anti–E-cadherin	Cell Signaling Technology	Cat# 24E10; RRID: AB_3698875
Anti-IRF1	ProteinTech	Cat# 11335-1-AP; RRID: AB_2877759

**Biological samples**		

Human placental tissue (22–28 weeks gestation)	Tokyo Medical University Hospital	Ethics approval: T2021-0257
Human placental tissue (second trimester)	Kyushu University	Ethics approval: 571-00

**Chemicals, peptides, and recombinant proteins**		

Forskolin	Cayman Chemical	Cat# 11018
Rotenone	Fujifilm Wako	Cat# P-056N
Orludostat	Selleck Chemicals	Cat# S8847
Brequinar	Selleck Chemicals	Cat# S6626
Oleic acid	Fujifilm Wako	Cat# 151-03425
Uridine	Sigma-Aldrich	Cat# U3003

**Critical commercial assays**		

RNeasy Mini Kit	Qiagen	Cat# 74106
TruSeq Stranded Total RNA LT Kit	Illumina	Cat# RS-122-2201
ReverTra Ace qPCR RT Kit	Toyobo	Cat# FSQ-201
PowerUp SYBR Green Master Mix	Thermo Fisher Scientific	Cat# A25742
SimpleChIP Plus Enzymatic ChIP Kit	Cell Signaling Technology	Cat# 9005
Nano-Glo Endurazine Substrate	Promega	Cat# N2590
LipiORDER	Funakoshi	Cat# FDV-0041
sFLT1 ELISA Kit	R&D Systems	Cat# DVR100C

**Recombinant DNA**		

pLKO.1-shSCR	Addgene	addgene #17920
pLKO.1 - TRC cloning vector	Addgene	Addgene #10878
pLV-IFITM1	VectorBuilder	N/A
pLV-IFITM2	VectorBuilder	N/A
pLV-IFITM3	VectorBuilder	N/A
pLV-NR2F1	VectorBuilder	N/A
pLV-ZFN554	VectorBuilder	N/A

**Experimental models: Cell lines**		

BeWo	JCRB Cell Bank	JCRB No. JCRB9111; RRID: CVCL_0044
HTR8/SVneo	Gift from Dr. Charles Graham	RRID: CVCL_7162

**Software and algorithms**		

STAR	Dobin et al.	RRID: SCR_015899
RSEM	Li and Dewey	RRID: SCR_013027
edgeR	Bioconductor	RRID: SCR_012802
Enrichr	Ma’ayan Lab	RRID: SCR_001575
R	R Foundation	RRID: SCR_001905
ImageJ	NIH	RRID: SCR_003070
UCSC Genome Browser	UCSC	RRID: SCR_005780

### Experimental model and study participant details

#### Human subjects

Human placental tissues were obtained from pregnant women at 22–28 weeks of gestation, including patients with hypertensive disorders of pregnancy (HDPs; *n* = 5) and patients with premature delivery without HDPs (Ctrl; *n* = 5). All patients in the HDP group were diagnosed with early-onset hypertensive disorders of pregnancy before 20 weeks of gestation. The maternal age ranged from 32 to 44 years. Gestational age at delivery ranged from 22 + 2 to 27+4 weeks, with birth weights between 338 g and 798 g. Four of the five neonates were male, and one was female. All cases were classified as severe early-onset HDP, including one case complicated by HELLP syndrome. Fetal growth restriction (FGR) was observed in two cases. Relevant maternal comorbidities included kidney transplantation in one case and adenomyosis in another, while the remaining cases had no notable comorbidities. The control group consisted of preterm deliveries without HDP. In this group, maternal age ranged from 24 to 40 years. Gestational age at delivery ranged from 22 + 6 to 25+0 weeks, with birth weights between 425 g and 712 g. Three neonates were male and two were female.

All participants provided written informed consent prior to sample collection. All subjects were female. The study was conducted in accordance with the Declaration of Helsinki.

The collection and use of human placental tissues for RNA sequencing and immunohistochemical analyses were approved by the institutional ethics committees of Tokyo Medical University (approval no. T2021-0257 and 1512), Tokyo University of Pharmacy and Life Sciences (approval no. 2023-008), and Kyushu University (approval no. 571-00).

#### Cell lines

BeWo human choriocarcinoma cells were obtained from the Japanese Collection of Research Bioresources (JCRB) Cell Bank (JCRB No. JCRB9011; RRID: CVCL_0044). BeWo cells are of female origin. Cells were maintained according to the supplier’s recommendations. HTR8/SVneo human extravillous trophoblast cells were kindly provided by Dr. Charles Graham (Queen’s University, Kingston, ON, Canada; RRID: CVCL_7162). HTR8/SVneo cells are of female origin and were maintained under standard cell culture conditions.

### Method details

#### RNA-sequencing (RNA-Seq), GO, and pathway analyses

Placental tissue was obtained from pregnant women at 22–28 weeks of gestation, including patients with HDPs (*n* = 5) and premature delivery (*n* = 5); informed consent was obtained, and approval was provided by Tokyo Medical University Hospital. The study was approved by the institutional ethics committees of Tokyo Medical University (T2021-0257) and Tokyo University of Pharmacy and Life Sciences (2023-008). For RNA-seq analysis, RNA was extracted using a RNeasy Mini Kit (Qiagen, Tokyo, Japan) according to the manufacturer’s instructions. High-throughput sequencing libraries were prepared using a TruSeq Stranded Total RNA LT Sample Prep Kit (Illumina, San Diego, CA, USA) according to the manufacturer’s instructions, and data analysis was performed by Macrogen Japan (Kyoto, Japan). Primary sequence data were deposited in the DDBJ (DNA DataBank of Japan) Sequence Read Archive (https://www.ddbj.nig.ac.jp/dra/index-e.html; accession number: DRA021720-021721). Data analysis was performed as described previously. Briefly, trimmed sequences were analyzed using the STAR/RSEM/edgeR pipeline, the human genome (hg38), and reference annotations obtained from the UCSC genome browser (https://genome.ucsc.edu). Significantly differentially expressed genes (DEGs) were identified on the basis of counts per million (CPM) levels. GO and enriched signaling pathway analyses were performed using the Enrichr tool (http://amp.pharm.mssm.edu/Enrichr/).

#### Cell culture

The BeWo human choriocarcinoma cell line (JCRB Cell Bank, Osaka, Japan) was grown in a 1:1 mixture of Ham’s F12 medium and Dulbecco’s modified Eagle’s medium (DMEM; Fujifilm Wako Pure Chemical Corp., Osaka, Japan) supplemented with 10% fetal bovine serum (FBS, Nichirei Biosciences, Tokyo, Japan) and 1% PSN (100 μg/mL penicillin and100 μg/mL streptomycin,; Thermo Fisher Scientific, Waltham, MA, USA) at 37 °C in humidified air containing 5% CO_2_. To induce syncytialization, the cells were treated with forskolin (FSK; 2.5 μM, Cayman Chemical, Ann Arbor, MI, USA), an adenylate cyclase activator, for 48 h. Additional treatments, including rotenone (Rote, 50 nM, Fujifilm Wako Pure Chemical Corp.), orludostat (Orlu; 1 nM, Selleck Chemicals, TX, USA), brequinar (Bre, 25 nM, Selleck Chemicals), oleic acid (OA; 10 μM, Fujifilm Wako Pure Chemical Corp.), and uridine (Uri, 100 μM, SIGMA-Aldrich, Tokyo, Japan) were administered for 48 h each. HTR8 cells were kindly provided by Dr. Charles Graham (Queen’s University, Kingston, ON, Canada) and were cultured in an RPMI 1640 medium (Fujifilm Wako Pure Chemical Corp), supplemented with 10% fetal bovine serum (FBS, Nichirei Biosciences) and 1% PSN (100 μg/mL penicillin and100 μg/mL streptomycin, Thermo Fisher Scientific) at 37 °C in humidified air containing 5% CO_2_.

#### RNA extraction and quantitative RT‒PCR

Total RNA was extracted using a RNeasy Mini Kit (Qiagen) according to the manufacturer’s instructions. Reverse transcription was performed using a ReverTra Ace qPCR RT Kit (Toyobo, Osaka, Japan) at 37°C for 15 min followed by 85°C for 5 min to synthesize cDNA, and the resulting cDNA was subjected to quantitative PCR (qPCR) using PowerUP SYBR Green Master Mix (Thermo Fisher Scientific, Waltham, MA, USA). The amplification parameters consisted of an initial denaturation at 95°C for 30 s, followed by 40 cycles of denaturation at 95°C for 5 s and annealing/extension at 60°C for 10 s.The sequences of primers used are listed in Table S1. Calibration curves were used to determine the amplification of each target gene with respect to the expression of a reference gene, glyceraldehyde-3-phosphate dehydrogenase (GAPDH). The mean crossing threshold (Ct) values for each target were calculated using Sequence Detection System software v2.3 (Thermo Fisher Scientific).^56^

#### Cell fusion assays

Cell fusion in BeWo cells was assessed using two complementary methods: an immunofluorescence-based assay and a NanoLuc-based HiBiT complementation system.

For the immunofluorescence assay, BeWo cells were fixed with methanol and incubated with an anti-E-cadherin antibody (1:200, #3195; Cell Signaling Technology, Tokyo, Japan) followed by an AlexaFluor 594-conjugated goat anti-mouse secondary antibody (Thermo Fisher Scientific) and AlexaFluor 488-conjugated goat anti-mouse secondary antibody (Thermo Fisher Scientific) to visualize the cell membranes. Nuclei were counterstained with 4′,6-diamino-2-phenylindole 2HCl (DAPI). Stained cell membranes and nuclei were observed using a BZ-X810 fluorescence microscope (Keyence, Osaka, Japan). Multinucleated cells were counted in five randomly selected microscopic fields per sample across three independent experiments.^57^ In the same fields, the number of nuclei within the syncytiotrophoblasts and the total number of nuclei were recorded, and the fusion index was calculated as follows:

Fusionindex(%)=(numberofnucleiinsyncytia/totalnumberofnuclei)×100

across three independent experiments.^58^ The data are presented as ratios relative to the control and shown as mean ± SEM from three independent experiments.

For the HiBiT-based fusion assay, BeWo cells were transfected with plasmids encoding either GFP-tagged LgBiT or mCherry-tagged HiBiT. Stable cell lines were generated through puromycin selection, yielding GFP-LgBiT- and mCherry-HiBiT-expressing BeWo cells. For the fusion assay, equal numbers of these cell types were cocultured for 24 h, followed by treatment with 2.5 μM FSK for 48 h to induce syncytialization. Luciferase activity, which is indicative of cell fusion, was measured using the Nano-Glo Endurazine Live Cell Substrate (Promega, Madison, WI, USA) according to the manufacturer’s instructions.^59^ The data are presented as ratios relative to the control and shown as mean ± SEM from three independent experiments.

#### Western blotting

Harvested cells were lysed in RIPA buffer (Thermo Fisher Scientific), followed by centrifugation at 15,000 × g for 3 min at 4°C. The protein concentration of the resulting supernatant was determined using the Bradford assay. Samples containing 15 μg of protein were mixed with 6× Sample Buffer (Nacalai Tesque) and subjected to SDS–PAGE using 12% Mini-PROTEAN TGX Gels and 10× Tris/Glycine/SDS Buffer (Bio-Rad Laboratories) at 200 V for 30 min. The separated proteins were transferred onto polyvinylidene difluoride (PVDF) membranes (Bio-Rad) using the Trans-Blot Turbo system (Bio-Rad) at 1.3 A and 25 V for 7 min.After blocking with Bullet Blocking One (Nacalai Tesque) for 5 min, the membranes were incubated overnight at 4°C with the following primary antibodies: anti-DHODH (1:2000, BC065245; ProteinTech), anti-IFITM1 (1:2000, BC000897; ProteinTech), anti-IFITM2 (1:2000, BC009696; ProteinTech), anti-IFITM3 (1:2000, BC006794; ProteinTech), anti-IRF1 (1:2000, BC009483; ProteinTech), anti-IRF3 (1:2000, EPR2418Y; ProteinTech), anti-phospho-IRF3 (*p*-IRF3, 1:2000, P15314; Abcam), and anti-SCD1 (1:2000, BC005807; ProteinTech).Following washes with Tris-buffered saline containing 0.1% Tween 20 (TBST), the membranes were incubated with horseradish peroxidase-labeled goat anti-rabbit or anti-mouse IgG (1:5000; Vector Laboratories) for 1 h at room temperature. Immunoreactive bands were detected using enhanced chemiluminescence (Merck Millipore) and the signals were captured using a C-DiGit Blot Scanner (LI-COR Biosciences). To normalize the signals, the membranes were stripped using a stripping solution (Fujifilm Wako Pure Chemical Corp.) for 30 min at room temperature and re-probed with an anti-GAPDH antibody (1:5000, 5A12; Fujifilm Wako) as an internal control. Densitometric analysis was performed using ImageJ software, and the protein expression levels were expressed as the target/GAPDH ratio.^60^

#### Lentiviral transduction for gene knockdown and overexpression

For gene knockdown, a short hairpin RNA (shRNA) targeting DHODH (sequence: 5′-GTGAGAGTTCTGGGCCATAAA-3′) was designed and cloned and inserted into a linearized pLKO.1 vector by PCR amplification using the following primers: forward, 5′-GGTGTTTCGTCCTTTCCACAAG-3’; reverse, 5′-TCGACCTCGAGACAAATGGCA-3’. PCR was performed using PrimeSTAR Max DNA Polymerase (Takara Bio, Shiga, Japan), and the amplified products were assembled with the vector using the NEBuilder HiFi DNA Assembly system (New England Biolabs Japan, Tokyo, Japan). A nontargeting control shRNA plasmid (pLKO.1-shSCR, Addgene plasmid #1864) was used as a negative control. Lentiviral particles were produced in Lenti-X 293T cells (Takara Bio) by cotransfecting the shRNA plasmid with Lentiviral High-Titer Packaging Mix (Takara Bio). The viral supernatants were collected, centrifuged to remove debris, filter sterilized, and stored at −80 °C. BeWo cells were transduced with the viral particles in the presence of 10 μg/mL polybrene (Nacalai Tesque) and selected with 3 μg/mL puromycin (Nacalai Tesque) for 2 days. Total RNA was collected after an additional 24 h of culture in puromycin-free medium.^61^

For gene overexpression, lentiviral vectors encoding human IFITM1, IFITM2, IFITM3, NR2F1, or ZFN554 (pLV-IFITM1, pLV-IFITM2, pLV-IFITM3, pLV-NR2F1, and pLV-ZFN554, respectively) were purchased from VectorBuilder Inc. (Chicago, USA). These vectors were packaged in Lenti-X 293T cells using the Lentiviral High Titer Packaging Mix (Takara Bio) and TransIT-293 Transfection Reagent (Takara Bio) following the manufacturer’s instructions. The resulting lentiviral supernatants were filtered and used to transduce BeWo cells in the presence of 10 μg/mL polybrene (Nacalai Tesque). After 48 h of infection, the cells were selected with 3 μg/mL puromycin for 2 days to establish stable overexpressing cell lines.^62^

#### Chromatin immunoprecipitation assay

To identify transcription factors that bind to specific regions of the *IFITM* promoter, candidate regulators of IFITM expression were extracted from the GeneCards database. In addition, potential transcription factor-binding sites within 3 kb upstream of the *IFITM* promoter were predicted using the JASPAR database (http://jaspar.genereg.net). Cultured cells were fixed with 1% formaldehyde for 20 min to cross-link protein‒DNA complexes. Chromatin was isolated and fragmented using the SimpleChIP Plus Enzymatic Chromatin IP Kit (Cell Signaling Technology) according to the manufacturer’s instructions. Immunoprecipitation was performed using antibodies against IRF1 (1:100; ProteinTech) or a rabbit IgG control (1:100; Cell Signaling Technology). The DNA fragments recovered from the immunoprecipitated chromatin were analyzed by real-time PCR with the primers listed in Table S2.

#### Immunofluorescence analysis

BeWo cells (4 × 10^3^) were seeded onto Ibidi μ-slides 8 well (Ibidi, Munich, Germany), fixed in cold methanol for 20 min and blocked with blocking buffer (Nacalai Tesque, Kyoto, Japan) for 1 h. The cells were incubated for 2 h with the following antibodies: rabbit anti-IRF1 (1:200), rabbit anti-E-cadherin (1:200), anti-IFITM1 (1:200), anti-IFITM2 (1:200), or anti-IFITM3 (1:200). The cells were subsequently with an AlexaFluor 594-conjugated rabbit or mouse antibody (1:100) (Thermo Fisher Scientific Inc., Tokyo, Japan) and with an AlexaFluor 488-conjugated rabbit or mouse antibody (1:100) (Thermo Fisher Scientific Inc.) for 1 h. The cells were stained with 4′,6-diamidino-2-phenylindole dihydrochloride (DAPI), mounted with anti-fade reagent (ProLong Glass Antifade Mountant, Thermo Fisher Scientific Inc.) and examined using a microscope (BZ-X810, Keyence).

#### Assessment of membrane fluidity

BeWo cells (4 × 10^3^) were seeded onto μ-Slide 6(VI) 0.4 (Ibidi) and cultured at 37°C for 48 h. Membrane fluidity was assessed via 1 μM LipiORDER (Funakoshi, Tokyo, Japan) following the manufacturer’s protocols. The fluorescence intensities of the liquid-ordered (Lo) phase (green) and liquid-disordered (Ld) phase (red) were measured at the cell-cell interface, and the Lo/Ld ratio was calculated as an indicator of membrane rigidity. To ensure quantitative accuracy, regions with pixel saturation were strictly excluded from the analysis. The fluorescence signals were detected using a BZ-X810 fluorescence microscope (Keyence) and ImageJ according to the manufacturer’s protocol.^63^ For each experiment, the fluorescence intensity corresponding to saturated fatty acids localized at the plasma membrane was quantified in three randomly selected fields per coverslip. Quantification was based on regions costained with E-cadherin, which was used as a membrane marker.

#### ELISA for sFLT1

The culture media were centrifuged at 10,000×g at 4°C for 10 min. The concentration of sFLT1 (sFLT1 Human VEGF R1/Flt-1 Immunoassay Kit, R&D System, Minneapolis, MN, USA) in the medium was quantified by ELISA, according to the manufacturer’s instructions.^64^

#### Invasion assay

The invasion ability of HTR8 cells was assessed using a transwell system (Chemotaxicell, Kurabo, Osaka, Japan) with polycarbonate membranes with an 8 μm pore size. IFITM-OE BeWo cells treated with FSK were seeded into the bottom wells of a 24-well culture plate. Transwell inserts precoated with Matrigel (Corning, NY, USA) were then placed in the wells, and HTR8 cells suspended in basal medium containing 2% FBS were added to the upper chambers. The lower chambers contained basal medium supplemented with 10% FBS to serve as a chemoattractant. After 24 h of incubation, the cells that invaded through the Matrigel and adhered to the underside of the membrane were fixed with cold methanol and stained with DAPI. Invaded cells were counted in five randomly selected microscopic fields per insert using ImageJ software (NIH, Bethesda, MD, USA).^65^

#### Immunofluorescence staining of tissue sections

Placental tissues were obtained from pregnant women in the second trimester of gestation, including patients with HDP (*n* = 3) and premature delivery (*n* = 3); informed consent was obtained, and approval was provided by Kyushu University. The study was approved by the institutional ethics committees of Kyushu University (#571-00) and Tokyo University of Pharmacy and Life Sciences (#1512). The tissue samples were immediately fixed in 4% paraformaldehyde in PBS, dehydrated, and embedded in paraffin. The sections were rehydrated and subjected to antigen retrieval by boiling in 10 mM citrate buffer (pH 6.0) for 20 min. The sections were incubated overnight at 4°C with the following primary antibodies: mouse polyclonal anti-IFITM2 (1:100), rabbit polyclonal anti-DHODH (1:100), and rabbit polyclonal anti-hCGB (1:100, BC022796, ProteinTech). After washing, the sections were incubated with horseradish peroxidase-labeled goat anti-rabbit or anti-mouse IgG (1:400; Vector Laboratories). Signal detection was enhanced using Tyramide Amplification Buffer Plus (Biotium, Fremont, CA, USA), with IFITM2 visualized in green, DHODH in red, and hCGB in white. Nuclei were counterstained with DAPI. Fluorescence images were captured using a BZ-X810 fluorescence microscope (Keyence). The fluorescence intensities of IFITM2 and DHODH within hCGB-positive regions were quantified in three randomly selected fields per section using ImageJ software (NIH).^62^

#### Statistical analysis

The data are presented as the means ± standard errors of the means (SEMs). Statistical comparisons were performed using Tukey’s test or an unpaired Student’s t test. A *p* value <0.05 was considered to indicate statistical significance. All analyses were conducted using R software (version 4.0.5; https://www.r-project.org).

For RNA-seq analyses, differential expression was considered statistically significant if the false discovery rate (FDR)-adjusted *p* value (q value) was <0.05, as specified in the text.
