# Supplementary material for: DHODH regulates trophoblast fusion via IFITM-reduced plasma membrane fluidity: Implications for hypertensive disorders of pregnancy
Source: iScience. 2026 Jun 8;29(6):116163. doi: 10.1016/j.isci.2026.116163 (PMC13264021; doi:10.1016/j.isci.2026.116163)
Supplement: Document S1. Figures S1–S4 and Tables S1 and S2 [file mmc1.pdf]

iScience, Volume *29*

Supplemental information

DHODH regulates trophoblast fusion via IFITM-reduced plasma membrane fluidity: Implications for hypertensive disorders of pregnancy

Kanoko Yoshida, Kazuya Kusama, Junya Kojima, Yu Kawaguchi, Kaito Suzuki, Tomoka Shimooki, Atsuya Tsuru, Mikihiro Yoshie, Masanori Ono, Hirotaka Nishi, Kiyoko Kato, and Kazuhiro Tamura

Table S1. Primers for real-time PCR analyses

Name (Accession No.)	Sequence			Product length (bp)
GAPDH	F: 5'-	AGCCACATCGCTCAGACA	-3'	66
NM_002046.7	R: 5'-	GCCCAATACGACCAAATCC	-3'	
sFlt1	F: 5'-	ACAATCGAGGTGAGCACTGCAA	-3'	180
NM_001159920.2	R: 5'-	TCCGAGCCTGAAAGTTAGCAA	-3'	
PlGF	F: 5'-	GAACGGCTCGTCAGAGGTG	-3'	188
NM_002632.6	R: 5'-	ACAGTTGCAGATTCTCATCGCC	-3'	
VEGFA	F: 5'-	TTGCCTTGCTGCTCTACCTCCA	-3'	126
NM_001025366.3	R: 5'-	GATGGCAGTAGCTGCGCTGATA	-3'	
DHODH	F: 5'-	GCCATAAATTCCGAAATCCAG	-3'	175
NM_001361.5	R: 5'-	ACAGCTTGGTCCTCAGGGAG	-3'	
OPA1	F: 5'-	GCTCTGCATACATCTGAAGAACA	-3'	52
NM_130835.3	R: 5'-	AGAGGCTGGACAAAAGACGTT	-3'	
DNM1L	F: 5'-	AAACTTCGGAGCTATGCGGT	-3'	66
NM_001278465.2	R: 5'-	AGGTTCGCCCAAAAGTCTCA	-3'	
MFN1	F: 5'-	TTGGAGCGGAGACTTAGCAT	-3'	71
NM_033540.3	R: 5'-	GCCTTCTTAGCCAGCACAAAG	-3'	
TFAM	F: 5'-	AGCTCAGAACCCAGATGCAA	-3'	238
NM_003201.3	R: 5'-	CCGCCCTATAAGCATCTTGA	-3'	
CGB	F: 5'-	CCTGGCCTTGTCTACCTCTT	-3'	108
NM_000737.3	R: 5'-	GGCTTTATACCTCGGGGTTG	-3'	
ERVFRD1	F: 5'-	CCAAATTCCCTCCTCTCCTC	-3'	115
NM_207582.2	R: 5'-	CGGGTGTTAGTTTGCTTGGT	-3'	
IFITM1	F: 5'-	ACTCCGTGAAGTCTAGGGACA	-3'	155
NM_003641.5	R: 5'-	TGTCACAGAGCCGAATACCAG	-3'	
IFITM2	F: 5'-	ATTGTGCAAACCTTCTCTCCTG	-3'	89
NM_006435.3	R: 5'-	ACCCCCAGCATAGCCACTTCCT	-3'	
IFITM3	F: 5'-	ACTGTCCAAACCTTCTTCTCTC	-3'	86
NM_021034.3	R: 5'-	AGCACAGCCACCTCGTGCTC	-3'	
NR2F1	F: 5'-	TGCCTCAAAGCCATCGTGCTGT	-3'	156
NM_001410754.1	R: 5'-	CAGCAGCAGTTTGCCAAAACGG	-3'	
ZFN554	F: 5'-	CCTCAAGCCTCCTGTTCAGATTGG	-3'	150
NM_001102651.2	R: 5'-	GCTGTCCTCTAACTGCTTCCATCC	-3'	
GCM1	F: 5'-	GCAACACCAACAACCACAAC	-3'	100
NM_003643.3	R: 5'-	GTAAATCTTGCGGCCTTCCT	-3'	

Table S2. Primers for real-time PCR analyses following ChIP assay

**Name**	**Sequence**	**Product length (bp)**
IFITM1	F: 5'- AGCCTCTCTCCTTAGCCTTCAG -3'<br>R: 5'- ACGGTGCTCAGTGAGACCTC -3'	250
IFITM2	F: 5'- AGGGAGCTCCATTCAGTCATG -3'<br>R: 5'- AGAATGTCCTGGGATCTGTGTC -3'	150
IFITM3	F: 5'- TCAGACCTCCTTCACTGTCATC -3'<br>R: 5'- AAGAAACTGCAGGGCACAAG -3'	112

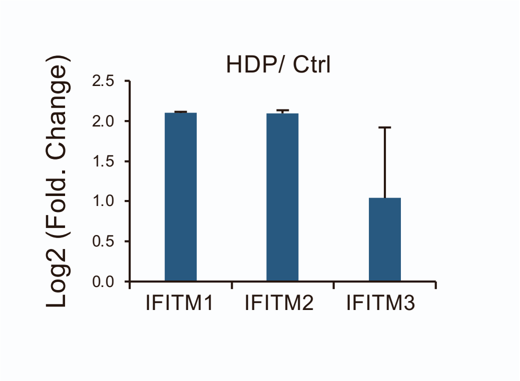

Figure S1. IFITM expression in RNA-seq using placenta with HDP

Expression of IFITMs in placental tissue was obtained from pregnant women at 22-28 weeks of gestation, including patients with hypertensive disorders of pregnancy (HDPs, n=5) and premature delivery (Ctrl, n=5) as measured by RNA-seq. The data are presented as the means ± SEMs.

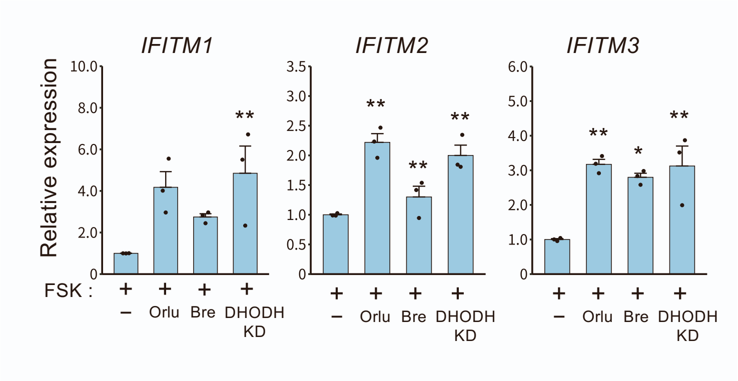

Figure S2. The effects of DHODH inhibition on the expression of IFITMs in trophoblast stem cell

Trophoblast stem cells or DHODH-knockdown(KD) trophoblast stem cells were treated with forskolin (FSK, 2.5 μM), orludodstat (Orlu, 1 nM), or brequinar (Bre, 25 nM) for 48 h. Expression of IFITMs in trophoblast stem cells as measured by qPCR. The data are presented as the means ± SEMs. *P < 0.05, **P < 0.01 vs. FSK alone.

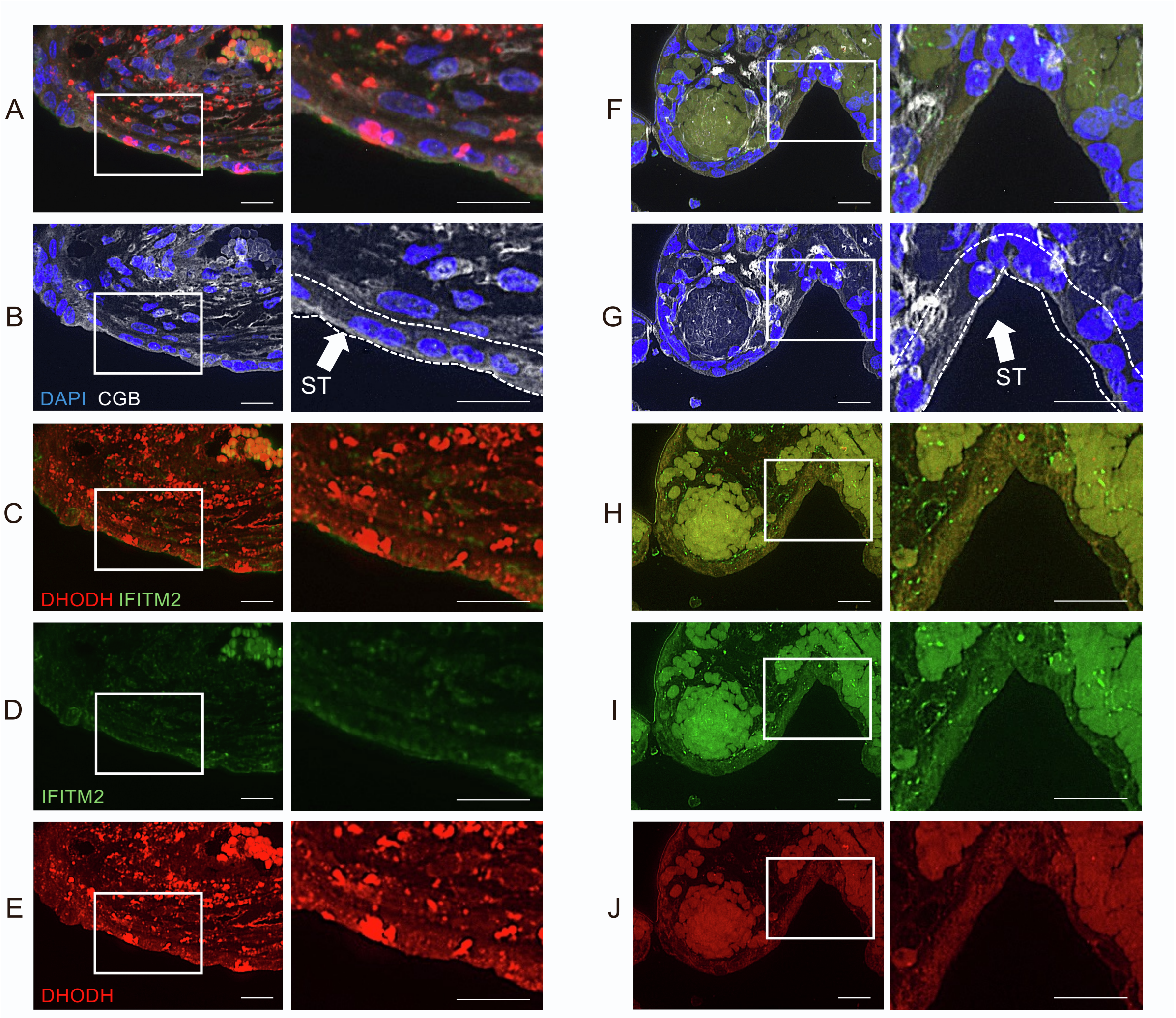

Figure S3. High-resolution images for Figure 8

Placental sections from the premature delivery (Ctrl, n=3), and HDP (n=3) were immunostained with anti-IFITM2 antibody (green), anti-DHODH antibody (red), anti-hCGB antibody (white, syncytiotrophoblasts), and DAPI (blue) to label nuclei. For each group, the right panel images are higher magnification views of the syncytiotrophoblast (ST) regions shown on the left panel. A representative image from three independent experiments is shown. Scale bar = 20 μm.

Figure S4. Uncropped Western blot images corresponding to each figure.

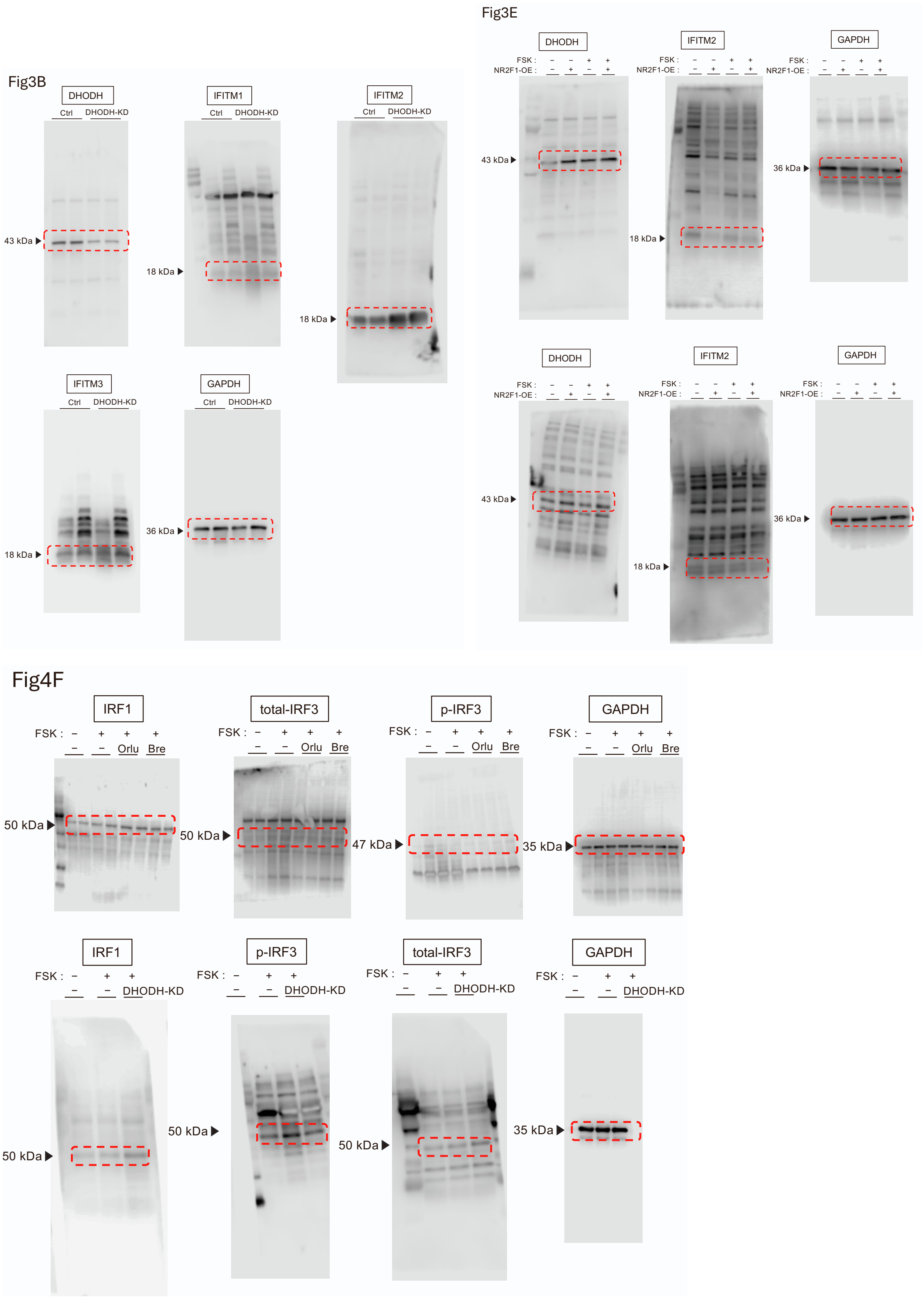

Fig5A

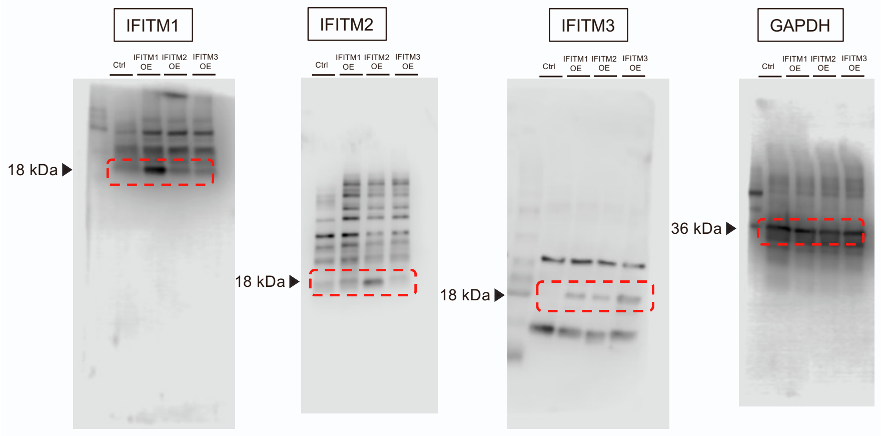
IFITM1
IFITM2
IFITM3
GAPDH
Ctrl
IFITM1 OE
IFITM2 OE
IFITM3 OE
18 kDa
18 kDa
18 kDa
36 kDa

Fig6B

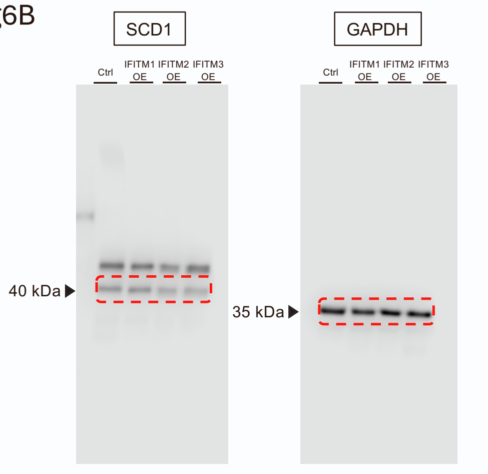
SCD1
GAPDH
Ctrl
IFITM1 OE
IFITM2 OE
IFITM3 OE
40 kDa
35 kDa
